# Food Sources for *Ruditapes philippinarum* in a Coastal Lagoon Determined by Mass Balance and Stable Isotope Approaches

**DOI:** 10.1371/journal.pone.0086732

**Published:** 2014-01-28

**Authors:** Tomohiro Komorita, Rumiko Kajihara, Hiroaki Tsutsumi, Seiichiro Shibanuma, Toshiro Yamada, Shigeru Montani

**Affiliations:** 1 Graduate School of Environmental Science, Hokkaido University, Sapporo, Japan; 2 Faculty of Environmental and Symbiotic Science, Prefectural University of Kumamoto, Tsukide, Kumamoto, Japan; 3 Nishimuragumi Co. Ltd., Hokkaido, Japan; CSIR- National institute of oceanography, India

## Abstract

The relationship between the food demand of a clam population (*Ruditapes philippinarum* (Adams & Reeve 1850)) and the isotopic contributions of potential food sources (phytoplankton, benthic diatoms, and organic matter derived from the sediment surface, seagrass, and seaweeds) to the clam diet were investigated. In particular, we investigated the manner in which dense patches of clams with high secondary productivity are sustained in a coastal lagoon ecosystem (Hichirippu Lagoon) in Hokkaido, Japan. Clam feeding behavior should affect material circulation in this lagoon owing to their high secondary productivity (ca. 130 g C m^−2^ yr^−1^). Phytoplankton were initially found to constitute 14–77% of the clam diet, although phytoplankton nitrogen content (1.79–4.48 kmol N) and the food demand of the clam (16.2 kmol N d^–1^) suggest that phytoplankton can constitute only up to 28% of clam dietary demands. However, use of isotopic signatures alone may be misleading. For example, the contribution of microphytobenthos (MPB) were estimated to be 0–68% on the basis of isotopic signatures but was subsequently shown to be 35±13% (mean ± S.D.) and 64±4% (mean ± S.D.) on the basis of phytoplankton biomass and clam food demand respectively, suggesting that MPB are the primary food source for clams. Thus, in the present study, the abundant MPB in the subtidal area appear to be a key food source for clams, suggesting that these MPB may sustain the high secondary production of the clam.

## Introduction

Suspension-feeding bivalves occurring in coastal waters and on tidal flats often establish high-density populations with extremely large standing stocks of biomass (i.e. over several kilograms in wet weight per square meter) and exhibit much higher productivity than other common members of macrobenthic communities. For example, the annual secondary productivity of dense patches of mussels, *Mytilus edulis* (L. 1758), reached 11 MJ m^−2^ yr^−1^ (275 g C m^−2^ yr^−1^, using 0.025 g C KJ^–1^ based on Brey [1]) in the sublittoral zone at Bellevue, Newfoundland, Canada [2], whereas that of surf clams (*Donax serra* (Röding 1798)) inhabiting the highly exposed sandy beaches of Namibia was reported to be 167–637 g ash free dry mass (AFDM) m^−2^ yr^−1^ (81.2–310 g C m^−2^ yr^−1^,using 0.486 g C gAFDM^–1^ based on Brey [1]) [3]. Moreover, the high secondary productivity of suspension-feeding bivalves corresponds to the range of primary productivity (165–320 g C m^−2^ yr^−1^ [4]–[6]) of microalgae that are suspended in the water in which the bivalves live, including phytoplankton and microphytobenthos (MPB); these microalgae are a primary source of food for the bivalves.

Based on the rate of conversion of food to somatic growth in invertebrates, which is typically assumed to be 20% [7], the amount of food consumed by suspension-feeding bivalves should be at least five times greater than the secondary productivity for the same population. Therefore, such bivalves require efficient mechanisms to allow the collection of sufficient food from the surrounding environment to sustain their high secondary productivities. However, the relationship between the secondary productivity of suspension-feeding bivalves and the availability of primarily produced organic matter such as phytoplankton and MPB remains poorly understood [8], [9].

Stable carbon and nitrogen isotope ratios indicate that suspension-feeding bivalves typically feed on primarily produced organic matter (such as phytoplankton, detritus, and resuspended MPB) suspended in water [10]–[12]. The resuspension of MPB and particulate matter derived from sediments have been observed to consistently occur shortly after maximum current velocity has been reached in muddy tidal flats, witch typically exhibit a relatively low threshold current velocity (ca. 15 cm s^–1^) [13]. The short-necked clam, *Ruditapes philippinarum* (Adams & Reeve 1850) as a junior synonym of *Tapes philippinarum*, is one of the most abundant suspension-feeding bivalves on sandy tidal flats along the Japanese coast [14]. The diet of this clam exhibit a spatial gradient that is primarily driven by tidal hydrodynamics within bays and by land-use characteristics within catchments [15], both of which can effect changes in the food supply [16]. Thus, the relative contributions of phytoplankton and MPB to the diet of *R. philippinarum* may simply be dependent on their relative availability [16]–[18].

The proportional contribution of different food sources to the diet of organisms is typically determined using only isotopic mass balance, which assesses the quality of diet components. Quantitative estimation of the food demand of dense patches of suspension-feeding bivalves has rarely been attempted with reference to the contributions of different food sources (i.e. the quantity of diet components). However, adopting such a quantitative approach is important for the precise estimation of material circulation via suspension-feeding bivalves, and this can allow a more realistic determination of the contribution of each food source.

The present study was conducted as part of a long-term research project that aimed to quantify population dynamics and associated energy and/or material flows at various trophic levels in the coastal lagoon ecosystem of Hichirippu Lagoon, located in the eastern part of Hokkaido, northern Japan. On the tidal flats of this lagoon, *R. philippinarum* constitutes one of the dominant macrobenthic communities, and its biomass often exceeds 265 g dry weight (DW) m^−2^ (approximately 5 kg wet weight (WW) m^−2^ using 0.053 gDW gWW^–1^) [19]. Abundant primarily produced organic matter is required to sustain the secondary productivity of dense patches of the clam; such matter is available for suspension feeding in the water overlying the sediment. Our recent study revealed that the areal biomass of MPB occurring in the surface layer of the sediment (i.e. within 0.5 cm of the surface) is about 100 times greater than the biomass of phytoplankton contained in the water column up to a depth of about 1 m during high tide in summer [19]. Therefore, both phytoplankton and MPB are likely available as primary food resources for clams; however, their availability varies seasonally. Moreover, through their suspension-feeding activities, clams exert a considerable influence on the flow of energy and materials between the primary (i.e. the microalgae) and dominant secondary (i.e. the clams themselves) producers in the tidal flat ecosystem.

In the present study, field surveys to monitor the physicochemical environmental conditions of water and sediment and performed quantitative sampling of macrobenthic animals including dense patches of the clam, *R. philippinarum*, were conducted between February 2005 and April 2006. The observed seasonal fluctuations in the abundance and biomass of the macrobenthic organisms and estimate the secondary productivity of the clam population for the entire tidal flat area in the lagoon were described. Furthermore, we compare the food demand of the clams to the amount of potential food sources (phytoplankton, benthic diatoms, and organic matter derived from the sediment surface, seagrass, and seaweeds) and the stable carbon and nitrogen isotope ratios of the clams with those of potential food sources both quantitatively and qualitatively. Finally, how the dense patches and high secondary productivity of the clam population are sustained in the tidal flat ecosystem of Hichirippu Lagoon is discussed.

## Materials and Methods

### Study Area

**Figure 1:**
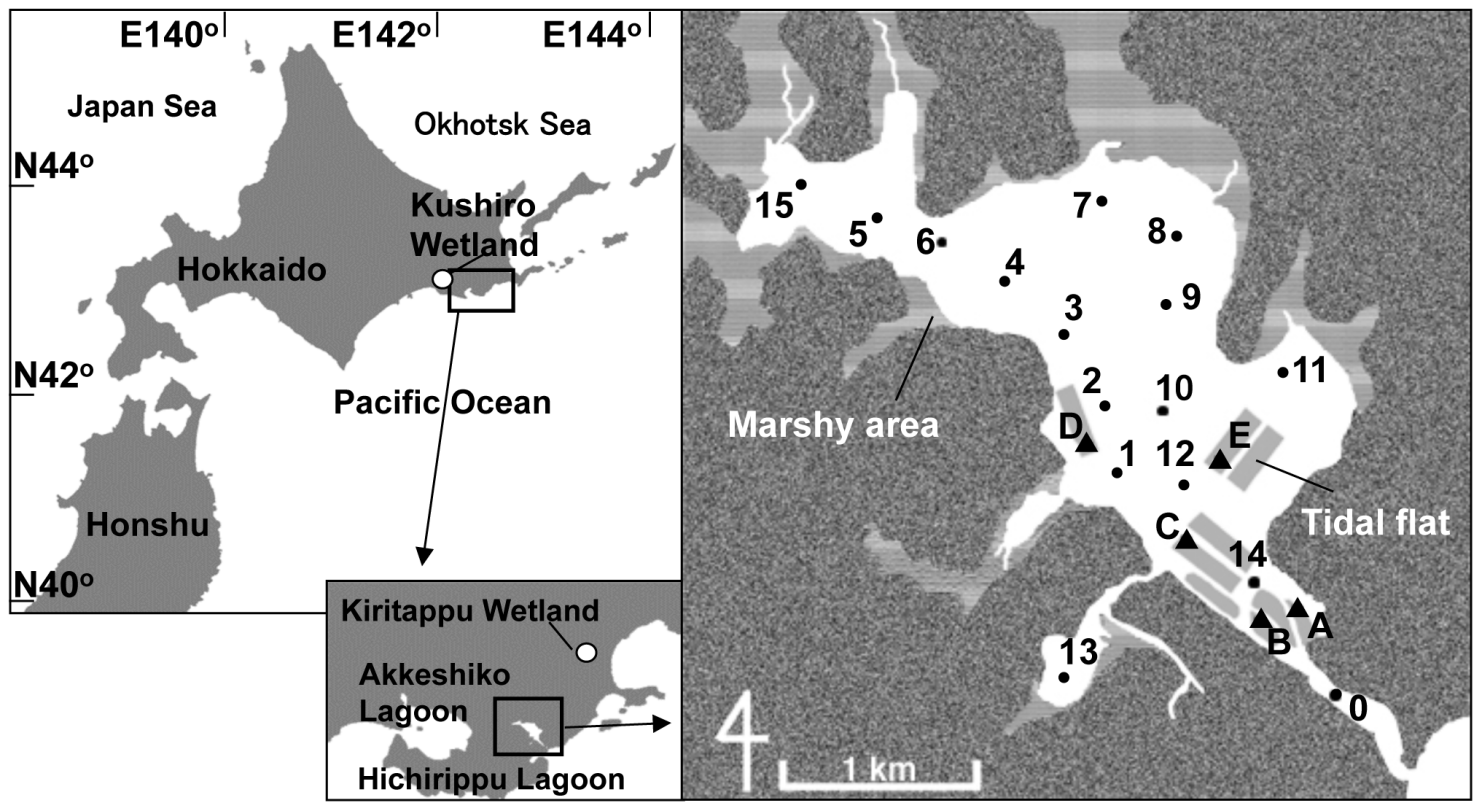
Study area and locations of sampling stations. The oval and rectangular grey areas represent naturally occurring (stations A and B) and artificial tidal flats (the other stations), respectively.

Hichirippu Lagoon bordering the Pacific Ocean in Hokkaido, Japan (44°03' N, 145°03' E, Figure 1) covers an area of approximately 3.56 km^2^ and is shallow (mean water depth: ∼1 m) and brackish. The maximum tidal flow at the entrance to the lagoon reaches approximately 40 cm s^−1^ during spring tide [19], and the flow direction is primarily from northwest to southeast. Freshwater has a relatively small effect on the water budget, ranging from 0.9 to 8.0% of total volume, and salinity ranges between 27.5–34.1 throughout the year [20]. Temperature of the surface sediment (approximately 5 cm depth) ranges from –9.5°C to 19.7°C at a tidal flat station [21]. The sediment in the subtidal area of the lagoon is muddy sand with the mud (less than 63 µm in particle diameter) content ranging between 8.7 and 28.1% [22]. At extreme low water spring tides, the area of the tidal flat exposed reaches approximately 0.19 km^2^; this represents around 5.3% of the total lagoon area. These tidal flats have typically been used as fishery sites to harvest the clam, *R philippinarum*, with 30–92 metric tons in wet weight of clam harvested per year during 1998–2004.

### Sampling Procedures

Five sampling stations on the tidal flats (stations A – E) to represent clam habitats and 16 stations (stations 0–15) in the subtidal area to represent a reservoir of food supply were selected (Figure 1). Quantitative sampling of macrobenthic animals were conducted and the concentration of chlorophyll-*a* (Chl-*a*) and organic matter in the surface sediment were measured at the tidal flat stations and Chl-*a* concentration in the water column and at the sediment surface were also monitored at the subtidal stations. This monitoring was conducted between February 2005 and April 2006, with monthly sampling from April to October and bimonthly sampling from December to April. Surface water samples were collected during flood tide. At each tidal flat station, sediment samples for geochemical analysis were randomly collected at 10 different sites within a 1 m radius of the station using an acrylic core tube (3 cm in diameter). The samples were extruded carefully and their surface layers (i.e. up to 0.5 cm depth) were retained to determine Chl-*a* and organic matter content of the sediment (SOM). At subtidal stations, sediment samples were carefully collected using an Ekman–Birge grab sampler, which sampled a 20×20 cm area to a depth of 20 cm. The topmost 0.5 cm of the sediment was also collected using an acrylic core tube.

For the quantitative survey of macrobenthic animals, 3–5 replicates of each sediment sample were collected using a stainless-steel core sampler (10×10×10 cm) and sieved through a 1 mm mesh screen. The residues of each sediment sample were stored in a plastic bag. Furthermore, 18–30 additional sediment samples were collected using the steel core sampler to improve the accuracy of the quantitative data describing the occurrence (in terms of density and biomass) of adult clams that were present at low densities. These additional samples were sieved through a 5 mm mesh screen and treated in the same manner as the other quantitative samples for macrobenthic animals.

From June to October 2005, 4 L of samples of surface water were collected in a sampling bucket at stations 0, 6, and 10 during flood tides to determine the stable carbon and nitrogen isotopic signatures of the suspended particulate organic matter (POM) in the water; these samples were stored in plastic bottles. The tidal flats near station 14 was selected as MPB sampling site, where locates on the center portion of the tidal flats. MPB samples were collected from the sediment surface by following a modified version of the method developed by Couch [23] for the determination of the stable isotopic signatures of carbon and nitrogen [22].

For quantitative analysis, macrophytobenthos (seagrass and macroalgae) were collected with bottom sediment using a handy core sampler (25×25×10 cm); sampling was performed five times for each station during August 2004 and October 2004. The samples were sieved through a mesh bag with a 1-mm opening and the residues of each sample in the mesh bag were stored in plastic bags. Seagrass (*Zostera japonica* L. 1753) and benthic macroalgae (*Ulva pertusa* L. 1753) were collected with the grab sampler in August 2005 and October 2005 for stable isotope analysis.

### Sample Treatment and Analysis

To determine the Chl-*a* content of the water, the water samples were filtered through glass fiber filters (Whatman GF/F) and Chl-*a* was extracted from residues on the filters in test tubes using 90% acetone. The test tubes were stored in darkness for 24 h at −20°C and then sonicated for 5 min. The concentrations of Chl-*a* in the supernatants were analyzed to estimate Chl-*a* standing stocks using a spectrophotometer (Turner 10-AU-5, Turner Designs) according to the method of Lorenzen [24]. To determine the Chl-*a* content of the surface sediment, Chl-*a* was extracted from duplicate subsamples of the wet surface sediment (∼0.5 g) in test tubes using 90% acetone. The concentrations of the supernatants were also determined by spectrophotometry [25].

Macrobenthic animals were sorted from the residues of the quantitative samples, identified, counted, and weighed by species. For the population study of *R. philippinarum*, the shell lengths of all specimens were measured using a digital caliper to produce a size–frequency distribution for the population. To determine the relationship between the shell length and dry weight of clam body tissues, 361 individuals were randomly collected in different seasons and their shell lengths were measured using the digital caliper. All of these clams were stored in filtered seawater for evacuation for 6–8 h. The body tissues were then removed from the shells, freeze-dried, and weighed. The relationship between the shell length and dry weight of body tissues for individual clams were obtained as follows:
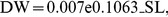
(1)


where r^2^ = 0.945, n  = 361, DW is dry weight, and SL is shell length.

The shell lengths of all specimens of the clams were measured for stable isotope analysis of carbon and nitrogen. The clams were then opened and their posterior adductor muscles were dissected, frozen at −20°C for storage, freeze-dried, and ground to powder with a mortar. Prior to analysis, the samples were treated with a chloroform–methanol mixture solution (2∶1, v/v) for 24 h to remove lipids, filtered with a precombusted GF/F filter (450°C, 5 h), rinsed with ethanol, and freeze-dried. The macroalgae and seagrass tissue used for stable isotope analysis were separated from the sample, rinsed with distilled water, freeze-dried, and ground into fine powder with a mortar. The organic matter derived from the surface sediment (SOM) was also ground into a powder with a mortar. Prior to analysis, the samples were treated with 1 N HCl to remove inorganic carbon, rinsed with deionized and distilled water to remove the acid, and freeze-dried. For the analysis of POM, 4 L of the water sample were filtered with a precombusted GF/F filter (450°C, 5 h), and the residues on the filter were used for stable isotope analysis. For the analysis of MPB, the MPB adhering to glass beads was rinsed with filtered seawater and sieved with a 125 µm mesh screen to remove the glass beads, which were then filtered using a precombusted GF/F filter (450°C, 5 h). Prior to analysis, the POM or MPB filters were treated with 1 N HCl to remove inorganic carbonates, rinsed with deionized and distilled water to remove the acid, and freeze-dried.

### Analysis of Stable Isotope Ratios of Carbon and Nitrogen

The stable isotope ratios of carbon and nitrogen were determined using a mass spectrometer (DELTA V Plus, Thermo Electron) connected directly to an elemental analyzer (Flash Elemental Analyzer 1112 Series, Thermo Electron). All isotopic data are reported in conventional delta notation (in ‰) as follows:
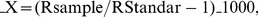
(2)where X is ^13^C or ^15^N and R is ^13^C/^12^C or ^15^N/^14^N for carbon and nitrogen, respectively. Pee Dee Belemnite and air N_2_ were used as the standards for carbon and nitrogen, respectively. The overall analytical error was within ±0.2 ‰.

### Estimation of the Secondary Production of the Clam Population

The shell lengths of all clams in the quantitative samples were measured using a digital caliper to obtain the size–frequency distribution of the clam population. Version 4.0 of the PROGEAN software [26] was used for generation analysis of the clam population by a graphic method. Mean shell length and the density of each cohort were determined from the size–frequency distribution of the population. Secondary productivity of the clam population was calculated in dry weight according to an incremental summation method [27] using density and mean shell length data for each cohort (obtained by generation analysis of the population) and the relationship between the shell length and biomass (in dry weight) of the clams (Equation 1).

P(*t*) represents the productivity of the clam population between sampling times *t* and (*t*−1) and is expressed as follows:
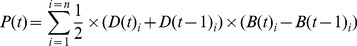
(3)where *i* is the cohort number, D(*t*)*i* is the density of cohort *i* at time *z t*, and B(*t*)*i* is the biomass of the individual with the modal shell size in cohort *i* at time *t*.

The estimated productivity of the clam population is represented by the amount of carbon and nitrogen based on the carbon and nitrogen contents of body tissues (40.1% and 12.3%, respectively), which allows evaluation of the importance of this productivity in material flow in the tidal-flat area.

### Data Analysis

POM from the lagoon, MPB, SOM, *U. pertusa*, and *Z. japonica* leaves were considered as potential trophic resources for the short-necked clam. The IsoSource software [28], [29] was used to determine the relative contribution of each source to the mixed signature of the clam adductor muscle at the tidal flats stations. The trophic enrichments of *R. philippinarum* (0.6 ‰ for δ^13^C and 3.4 ‰ for δ^15^N; [30]) were subtracted from the adductor muscle values before IsoSource analysis.

The carbon content (C/Chl) of phytoplankton and MPB were assumed to be 20–50 [31] and 10–150 [32], respectively, and the elemental compositions (C/N) of phytoplankton and MPB were assumed to be 6.6 [33] and 7.5 [25], respectively. The nitrogen content of *Z. japonica* and *U. pertusa* determined by the elemental analyzer were 2.8% and 1.9%, respectively. The standing stock of nitrogen in the microalgae was obtained by multiplying these elemental ratios with the microalgal standing stock.

#### Ethics statement

The field survey of this study is approved by the Chirippu Fishery Cooperative Union.

## Results

### Macrobenthic Community at the Representative Station

**Figure 2:**
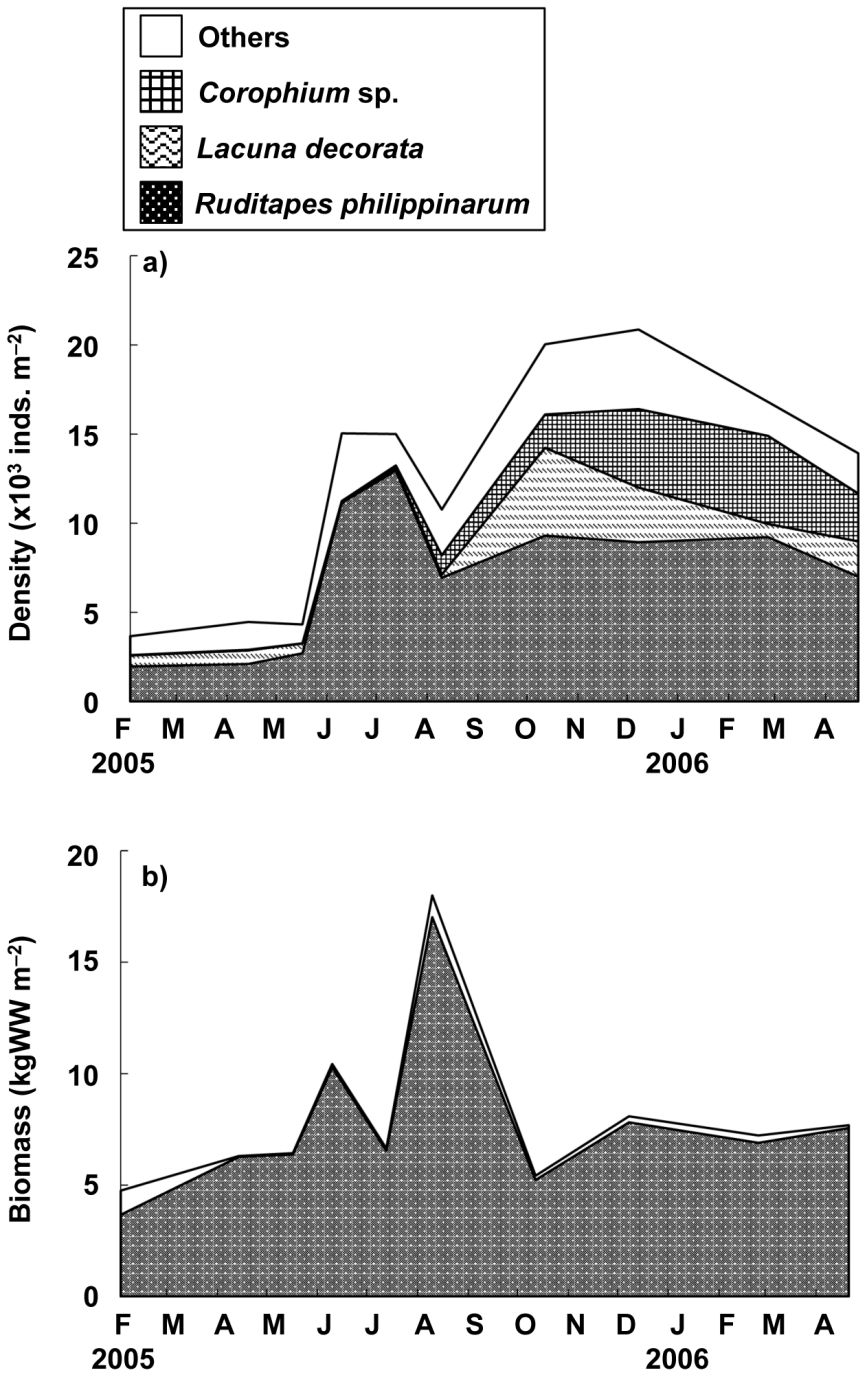
Seasonal variability of the macrobenthic animals at station B. (a) density and (b) biomass.

Station B was defined as a representative station for the macro-benthic animals, because it was suitable to analyze the cohort of the clam population at this station with the high density and biomass of the clam. Figure 2 illustrates the seasonal fluctuations in the density and biomass of macrobenthic animals at station B. The short-necked clam (*Ruditapes philippinarum*) was found to dominate the benthic community by density, accounting for 57.9% of all macrobenthic animals collected in the present study. The density of the clam peaked at 12960 ind. m^−2^ on July 19, 2005 and decreased gradually to 7000 ind. m^−2^ by April 27, 2006. A small gastropod, *Lacuna decorata* (Adams 1861), and an amphipod, *Corophium* sp., were the second and third most dominant species in the macrobenthic community. The density of *L. decorata* tended to increase in late summer to autumn and reached a peak density of 4930 ind. m^−2^ in October 2005 (Figure 2a). *Corophium* sp. also increased from 1,870 ind. m^−2^ to 4940 ind. m^−2^ between October 2005 and March 2006. In terms of biomass, *R. philippinarum* dominated the macrobenthic community exclusively (Figure 2b). The biomass of the clam was found to be 3.65–17.0 kgWW m^−2^, with a mean biomass of 7.77±3.68 kgWW m^−2^ (all values are given as mean ± SD), throughout the duration of the present study; this represents 95.9% of the total biomass in wet weight of the specimens collected.

### Biomass, Secondary Productivity, and P/B of *R. philippinarum*

**Figure 3:**
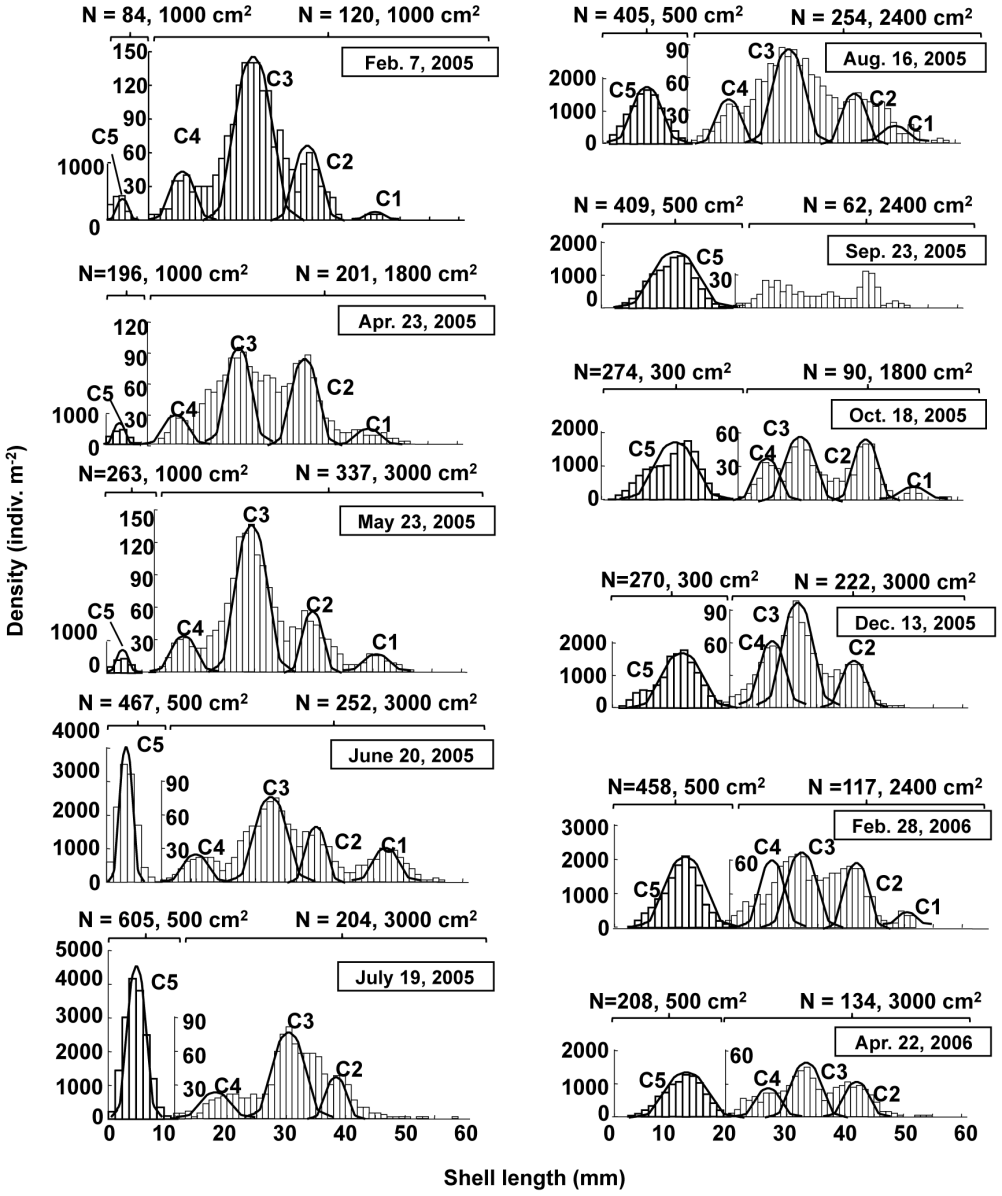
Size–frequency distribution of *Ruditapes philippinarum* population at station B. The left and right scales correspond to samples obtained using mesh sizes of 1 and 5(C = cohorts; n = number of individuals).

**Figure 4:**
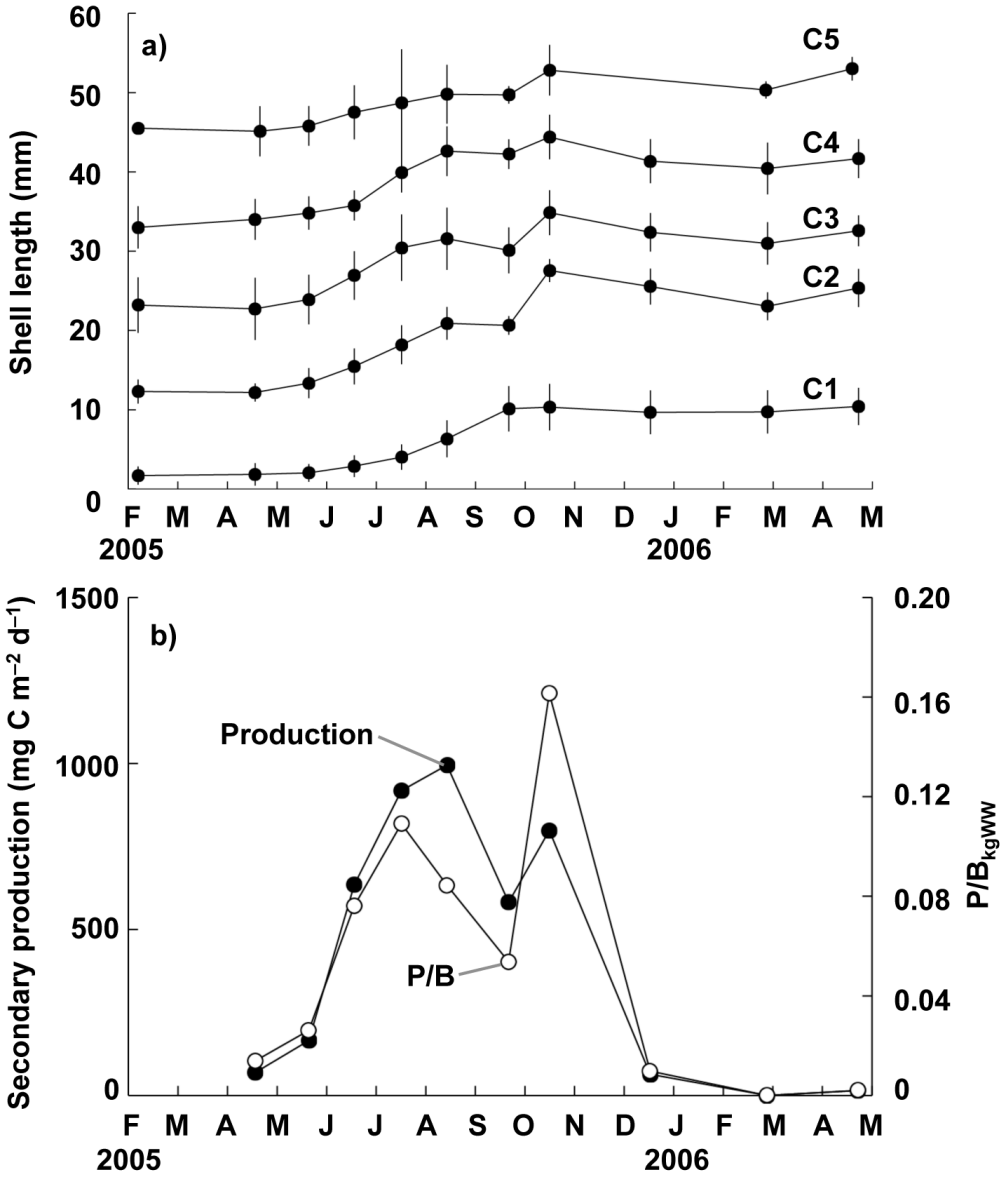
Seasonal variation of growth of the clam (*Ruditapes philippinarum*) at station B. Changes in (a) mean shell length of each cohort of the clam *Ruditapes philippinarum* and (b) daily secondary productivity and P/B_kgWW_ at station B.

The size–frequency distributions of the clam population between February 2005 and April 2006 are presented in Figure 3; these distributions were obtained by combining data collected from quantitative samples covering two different size ranges for macrobenthic organisms. The presence of five different cohorts (C1–C5) can be recognized in these size–frequency distributions, and the shell growth curves of these five cohorts are plotted in Figure 4a. In the study area, the clam breeds in September and planktonic larvae settle on tidal flat sediments in October [21]. In the size–frequency distributions, a new cohort (C5) initiated recruitment in April 2005, reaching a peak in recruitment in July 2005 with a density of 11250 ind. m^−2^. Moreover, all five cohorts recognized here exhibited increases in shell length of 5–12 mm in the warm season between May 2005 and October 2005. In contrast, their growth was extremely depressed or ceased altogether in the cold season between November 2005 and April 2006. From the growth curves produced, the clam was considered as approximately 1 year and 5 years to reach shell lengths of 10 mm and 50 mm, respectively, in the area investigated.

Figure 4b illustrates seasonal fluctuations in the secondary productivity of the clam population estimated from changes in the size–frequency distributions of the five cohorts. The annual secondary productivity of the clam population was 130 g C m^−2 ^yr^−1^ between April 2005 and April 2006, peaking at 994 mg C m^−2 ^d^−1^ in August 2005 and decreasing to 0–64.0 mg C m^−2^ d^−1^ between December 2005 and April 2006 owing to the depression or cessation of growth. Seasonal variation in the ratio of the daily secondary productivity of the clam to its biomass in kg wet weight (P/B_kgWW_) at each sampling instance exhibited a pattern similar to that of secondary productivity and varied between 0 and 0.16 (Figure 4b). The period between June and October was defined as the productive period, i.e. the period during which secondary production was greater than 500 mg C m^−2^ d^−1^.

**Figure 5:**
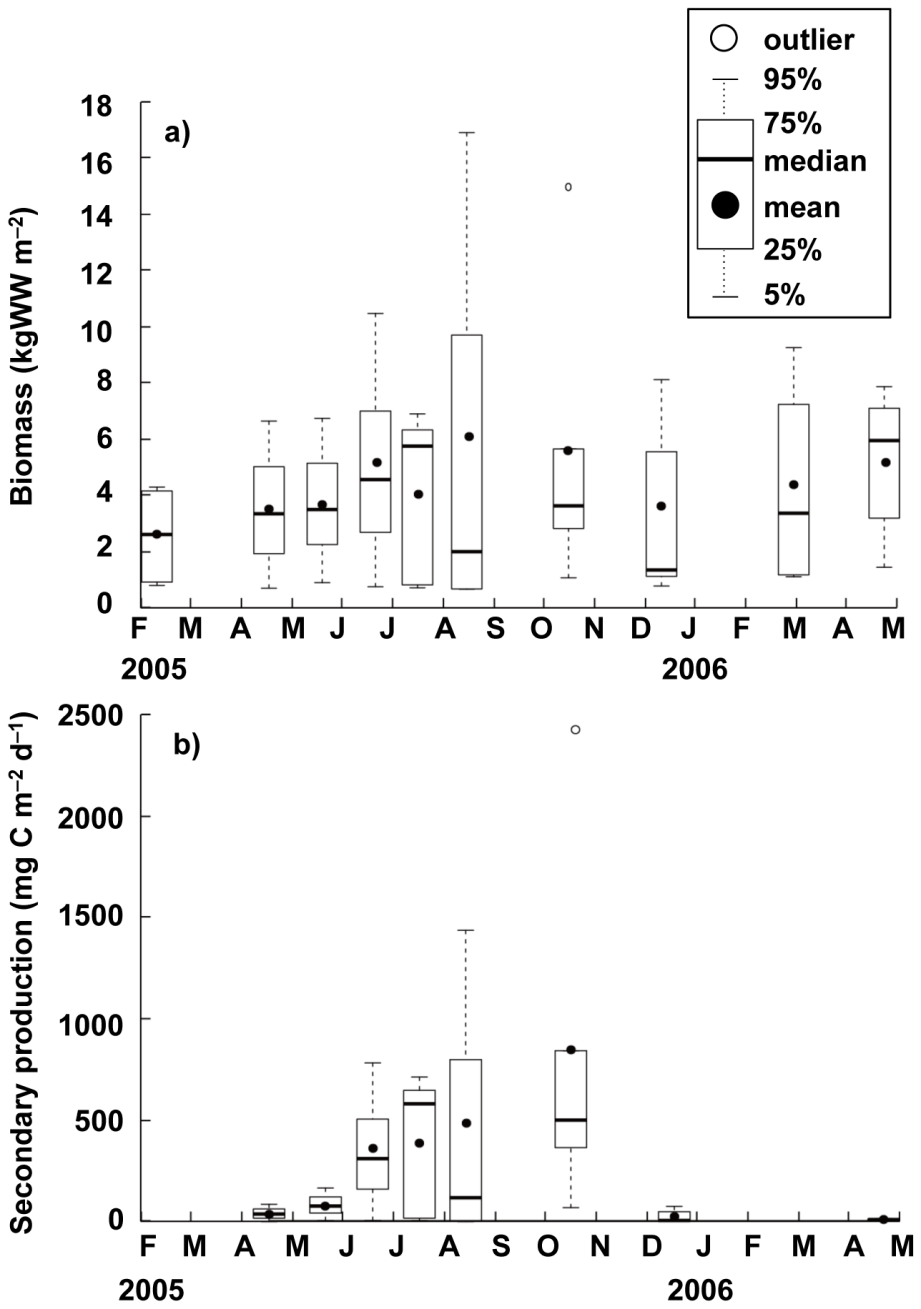
Seasonal variation of the clam (*Ruditapes philippinarum*) population at tidal flats stations. (a) biomass (kgWW m^–2^) and (b) secondary productivity (g C m^–2^ d^–1^) of the clam at five tidal flat stations, expressed as box plots.

Figure 5 illustrates seasonal variation in biomass and secondary productivity for the tidal-flat area. The biomass of clams was found to be heterogeneously distributed among tidal-flat stations (Figure S1); although, the clam accounted for at least 70% of the biomass of the benthic community. The mean values were found to be relatively stable, ranging from 1.98±1.77 kgWW m^−2^ to 5.59±7.51 kgWW m^−2^ (Figure 5a). The secondary productivity at the tidal-flat stations, calculated based on the mean biomass (Figure 5a) and P/B_kgWW_ (Figure 4b) at the representative station, exhibited a clear seasonal pattern: productivity peaked at 840±634 mg C m^−2^ d^−1^ in October 2005 and decreased to 9.6±5.9 and 28.0±33.8 mg C m^−2^ d^−1^ by December 2005 and April 2006, respectively. Thus, the annual secondary productivity was estimated to be 96.5 g C m^−2^ yr^−1^ throughout the tidal flats. During the productive period (i.e. for 120 days during June–October 2005), the integrated secondary productivity accounted for 90.6% (87.5 g C m^−2^) of the annual productivity.

### Food Demand of the Clam and Quantification of Potential Food Sources

Table 1 presents the net growth efficiency (i.e. growth/consumption) of six different species of bivalves including *R*. *philippinarum*. The food demand of the clam population (mg C m^−2^ d^−1^) was determined using an assimilation number of 18.8%, which was the mean value obtained from nine different energy budgets for the bivalve species (Table 1).

**Table 1:** Net growth efficiency (%) of bivalves.

Species	Country	Net growth efficiency	Method*	Reference
*Cardium edule*	Sweden	15.4	in situ+lab	[7]
*Mya arenaria*	Sweden	15.5	in situ+lab	[7]
*Macoma balthica*	Netherland	4.4	in situ+lab	[47]
*Ruditapes philippinarum*	USA	41.7	lab	[48]
*Scrobicularia plana*	Wales	14.8	in situ+lab	[49]
*Tellina tenuis*	Scotland	13.6	core	[50]
*Tellina tenuis*	Scotland	16.2	core	[50]
*Tellina tenuis*	Scotland	28.7	core	[50]
	Mean	18.8		

* lab: laboratory experiments, core: field core incubation experiments.

During the productive period, nitrogen-based secondary productivity and the food demand of the clam was 1.92 mol N m^−2^ and 10.2 mol N m^−2^, respectively, and the total secondary productivity and food demand of the clam throughout the tidal flats (i.e. for 0.19 km^2^) were 364 kmol N and 1939 kmol N, respectively. The daily secondary productivity and food demand of the clam during the productive season were 3.04 kmol N d^−1^ and 16.2 kmol N d^−1^, respectively, for the entire tidal flat area (0.19 km^2^) (Table 2).

**Table 2:** Food demand of the clam (*Ruditapes philippinarumm*) and total amount of potential food sources during the productive season.

Food demand of the clam	Region*	kmol N d^–1^	
	Tidal flats	16.2	
Amount of potential food sources		kmol N (min.–max.)	Amount/Demand (min.–max.)
Phytoplankton	Whole	1.79–4.48	0.11–0.28
Microphytobenthos	Tidal flats	6.8–16.9	0.42–1.04
	Subtidal	120–300	7.41–18.5
* Zostera japonica*	Subtidal	685	42.3
* Ulva pertusa*	Subtidal	31.1	1.92

Area: entire (3.56 km^2^), subtidal (3.37 km^2^), and tidal-flat (0.19 km^2^).

During the productive season, the mean biomass per unit area of MPB in the surface sediment (depth: 0.5 cm; 140.6±79.8 mg Chl-*a* m^−2^ in the subtidal area, n  = 56; 140.6±52.5 mg Chl-*a* m^−2^ on the tidal flats, n  = 39) was approximately 70 times greater than that of phytoplankton in the water column (depth: 1 m; 2.0±1.3 mg Chl-*a* m^−2^) (n  = 123). The mean standing stocks of *Zostera japonica* and *Ulva pertusa* were 98.8±102.4 g DW m^−2^ and 19.9±129.2 g DW m^−2^, respectively, in the subtidal area (n  = 30). The nitrogen-based biomasses of MPB, phytoplankton, *Z. japonica*, and *U. pertusa* were 213–3200 mmol N m^−2^ (for both the tidal flats and the subtidal area), 6.04–15.1, 203, and 9.22 mmol N m^−2^, respectively.

The total nitrogen content of phytoplankton (1.79–4.48 kmol N) was lower than that for all other organisms in the lagoon, and phytoplankton accounted for only 12–28% of the daily food demand of the clam population (Table 2). MPB in tidal flats also imposed limits on the daily food demand of the clams, accounting for 42–104% of the daily food demand. However, none of the other food sources studied imposed limits on the daily food demand of the clams.

### Carbon and Nitrogen Isotopic Signatures and Contribution to the Diet of the Clam

**Figure 6:**
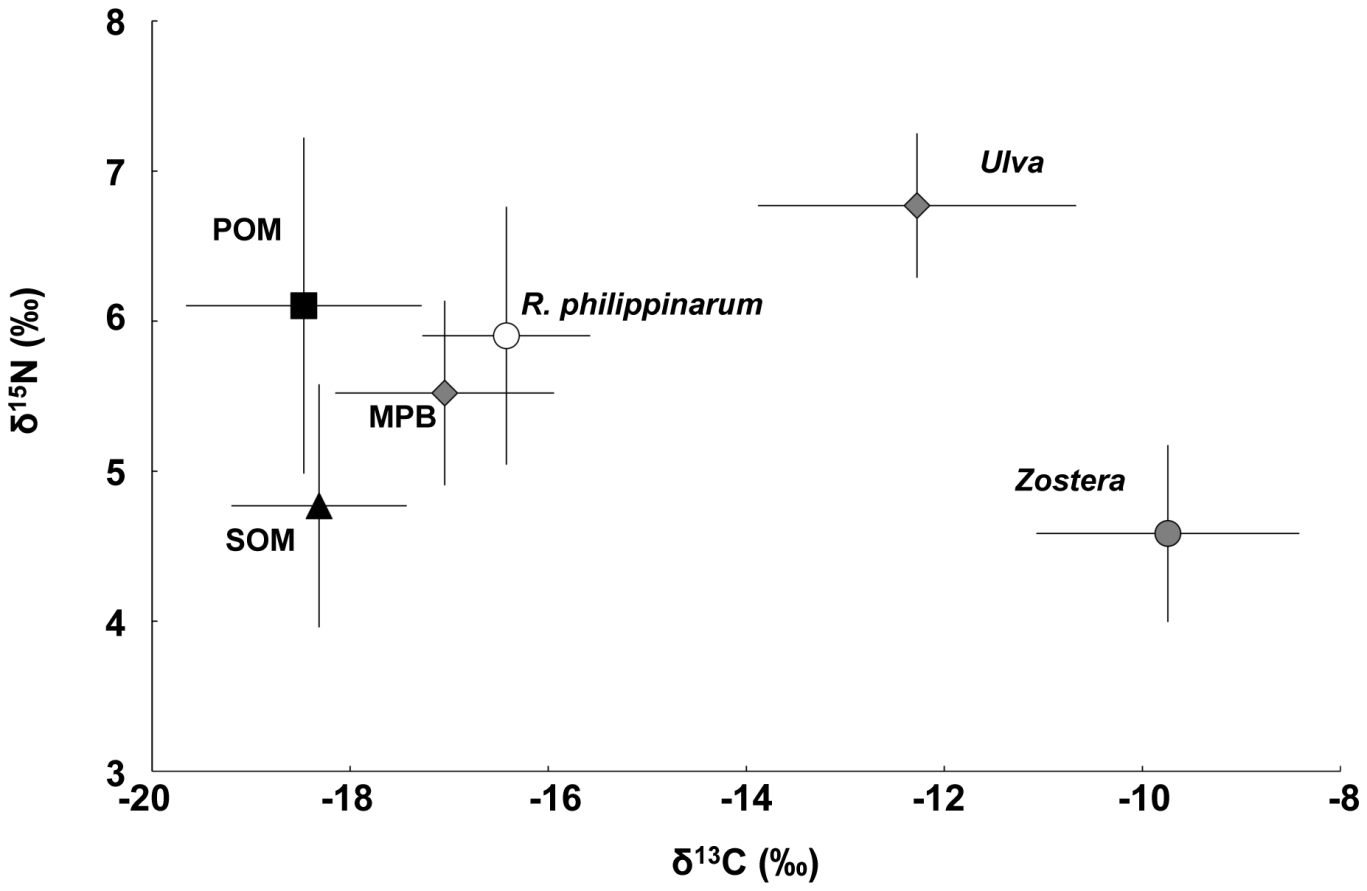
A dual isotope plot for carbon and nitrogen for the expected diet of the clam (*Ruditapes philippinarum*) and their potential food sources in Hichirippu Lagoon. See text in Materials and methods for diet-tissue fractionation for the clam to determine expected diet (*R. philippinarum*). Error bars are standard deviations.

The carbon stable isotope ratios (δ^13^C) of the clam diet and potential food sources in the lagoon ranged from −18.5±1.2 ‰ for POM to −9.7±0.6 ‰ for *Z. japonica*, whereas the nitrogen isotope ratios (δ^15^N) ranged from 4.8±0.9 ‰ for SOM to 6.8±0.5 ‰ for *U. pertusa* (Figure 6). Thus, the diet of the clam was found to exhibit −16.4±0.9 ‰ δ^13^C and 5.9±0.9 ‰ δ^15^N, which corresponds to the isotopic signature of MPB (−17.1±1.1 ‰ δ^13^C and 5.5±0.6 ‰ δ^15^N). At our study site, the δ^13^C value of POM fell within the ranges of previously reported for temperate marine phytoplankton (–24 ‰ to –18 ‰) from other estuarine and coastal waters [17], [34]–[36]. Thus, the isotope signature of the POM samples was regarded in this study to primarily reflect the presence of phytoplankton.

**Figure 7:**
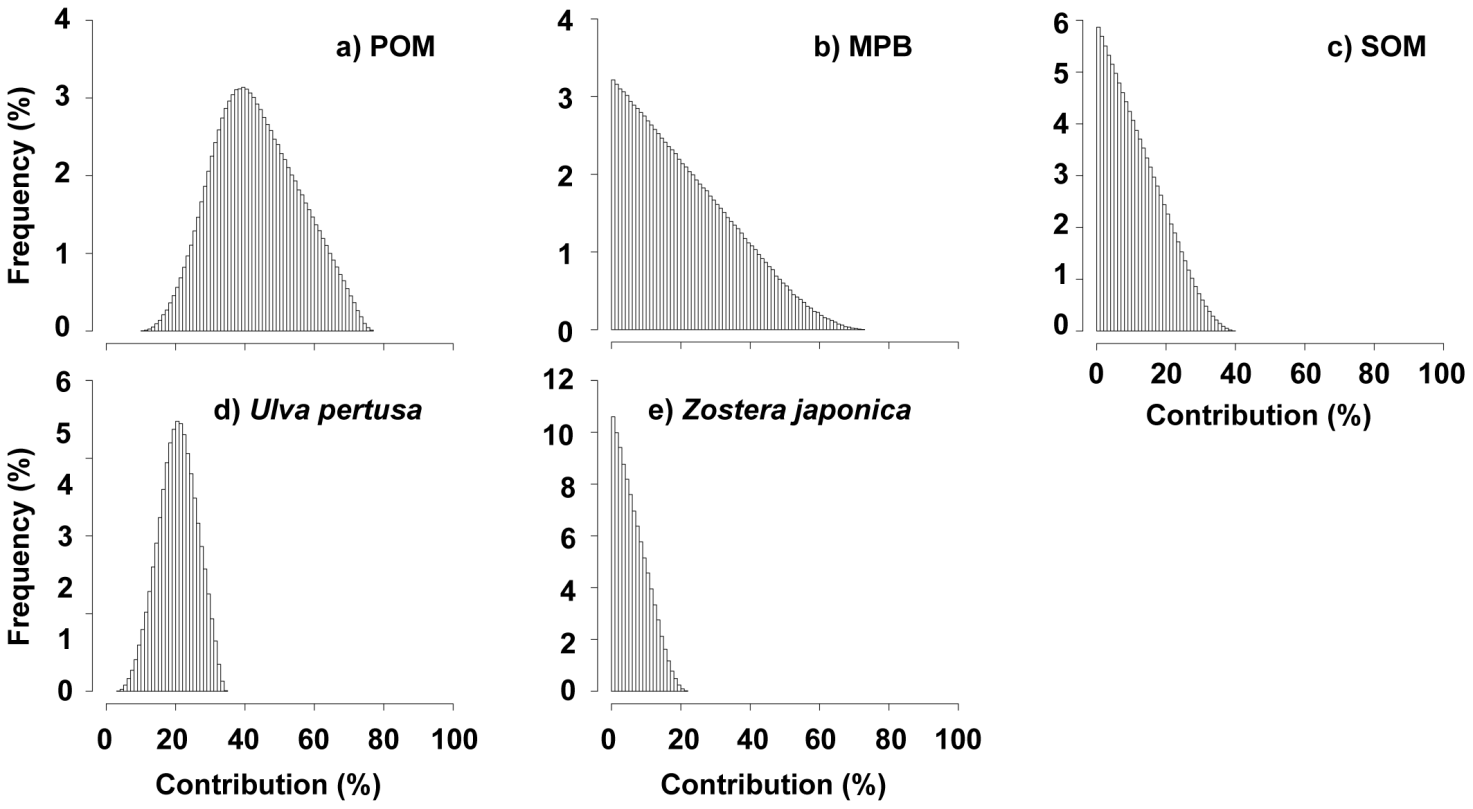
Histograms of the proportional contribution of each food source to the diet of the clam (*Ruditapes philippinarum*) based on the IsoSource mixing model.

According to the IsoSource mixing model, POM was the primary contributor to the clam diet (43±12%) (all values are given as mean ± SD) followed by MPB (20±15%) has a wider range (min.–max.: 0–69%) and *U. pertusa* (20±6%) has a narrower range (min.–max.: 3–34%); SOM, and *Z. japonica* contributed, on average, 11±8%, and 6±4%, respectively (Figure 7).

**Figure 8:**
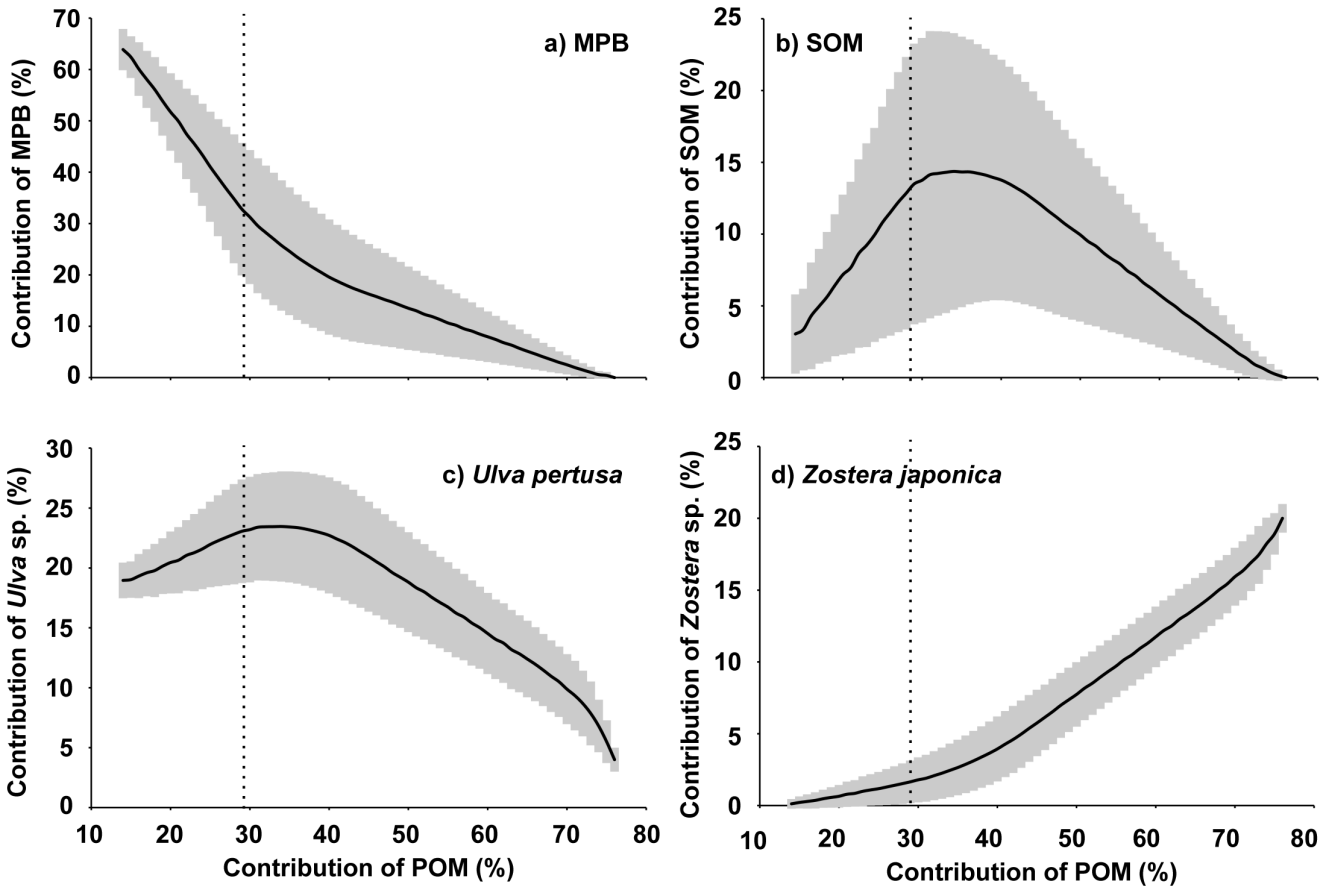
Scatter plot illustrating the relationship between the contributions of phytoplankton and other food sources. (a) MPB, (b) SOM, (c) *U. pertusa*, and (d) *Z. japonica*. Solid lines and grey region indicate mean and standard deviations for every 1% increase of POM contribution, respectively. Vertical dotted line indicates the upper limit to the phytoplankton contribution based on their abundance and the food demand of the clam.

Comparison of the total amount of phytoplankton present to the daily food demand of the clam indicates that phytoplankton account for 12–28% of the daily food demand of the clam (Table 2). A curve was constructed to a represent the mean contribution (and associated standard deviations) of potential food sources at each contribution (i.e. for every 1% increase) of POM, thus illustrating the entire range of solutions using only isotopic constraints (for all regions). Then, a subset of the output (left side of the dotted line) was extracted; this subset contains only solutions that satisfy the food demands of the clam population (Figure 8). For this subset, MPB was the primary contributor (35±13% to 64±4%), followed by *U. pertusa* (19±1% to 23±4%). The contribution of SOM and *Z. japonica* to the clam diet were 3±3% to 13±9% and 0±0% to 2±1%, respectively.

## Discussion

The short-necked clam (*R. philippinarum*) was found to be the dominant macrobenthic species in the present study area (Figure 2a, b). Comparison of the secondary productivity of the clam with that of the other suspension-feeding bivalve species present demonstrates that higher secondary productivity (i.e. 228–996 gC m^−2^ yr^−1^) was found in the following mytilid species: *Choromytilus meridionalis* (Krauss 1848) [37], *Perna picta* (Born 1778) [38], [39], and *M. edulis* [2]. These species typically form extremely dense populations, which accounts (at least primarily) for their high productivity. During the productive season, secondary productivity of the clam population in the lagoon was estimated to be 364 kmol N (per 120 days) based on both the mean secondary productivity of the clam and the area of the tidal flats (0.19 km^2^). A recent study showed that ingress of nitrate from the Pacific Ocean through the tidal inlet is necessary to maintain the lagoon ecosystem, and the dissolved inorganic nitrogen budget of this lagoon has been estimated to be 258 kmol N yr^−1^ [20]; this is comparable to the secondary productivity of the clam. Therefore, the feeding of the clam should have a considerable impact on the material circulation of the lagoon.

Yokoyama et al. [17] suggested that the relatively large biomass of phytoplankton and the scarcity of benthic microalgae could have produced the phytoplankton-based trophic structure (including *R. philippinarum*) found in Ariake Bay, southern Japan. In contrast, several authors have suggested that the clam depends more on MPB than on phytoplankton based on the biomass of microalgae [16], [18], [35]. In the present study, the relatively low contribution of the phytoplankton (at most 28%) as a food source for the clam should correspond to the occurrence of low phytoplankton biomass (Table 2); however, this is not the case. Although the contribution of the phytoplankton was estimated initially to be 14–77% based on isotopic data alone (Figure 7a), the stable isotope signatures obtained indicate that the phytoplankton contribute (at most) 28% of the food resources required for the clam population in the study area (Figure 8). Thus, the contributions derived using only isotopic signatures may be somewhat misleading.

Assuming a POM contribution of 28% based on the phytoplankton biomass, the clam population within the tidal flats (i.e. an area of 0.19 km^2^) must consume all of the phytoplankton present in the entire lagoon (i.e. an area of 3.56 km^2^) each day. Filtration experiments conducted on the clam [40] have demonstrated its weight-specific filtration rate for Chl-*a* is 2.3 L gDW^−1^ h^−1^ under temperatures of 24°C and mean Chl-*a* concentrations of 3.1 µg L^−1^. However, the temperature and Chl-*a* concentration for the water column in the present study were somewhat lower than those for the filtration experiment (Table 3) [20], [22]. Assuming that the weight-specific filtration rate of the clam (which has a mean biomass of 246–276 gDW m^−2^ in the study area) remains constant throughout the day, the filtration rate for clams on the tidal flats of the present study can be estimated to be 2.89–7.17 kg Chl-*a* d^−1^. The total amount of Chl-*a* in the lagoon was 3.56–9.90 kg; thus, the dense clam population appears to filtrate 72.5–80% of the Chl-*a* of water in the lagoon each day. The feeding activity of the clam population should have a considerable effect on the water column phytoplankton; however, it is highly unlikely that the clams present could consume all of the phytoplankton in the lagoon water owing to the circadian nature of suspension-feeding bivalves [41], [42]. Thus, it is reasonable to assume that the contribution of the phytoplankton to the clam diet is less than 28%.

**Table 3:** Estimation of the rate of filtration by the clam (*Ruditapes philippinarum*) in Hichirippu Lagoon during productive season.

	June	August	October
Clam biomass (gDW m^–2^)	246 (211)	296 (398)	276 (304)
Chl-*a* conc. (µg L^–1^)	2.78 (1.51)	1.49 (1.16)	1.00 (0.67)
Filteration rate (L gDW^–1^ h^–1^)	2.3		
Capacity of filteration(kg Chl-a d^–1^)	7.17	4.63	2.89
Amount of Chl-*a* (kg)	9.90	5.30	3.56
Filteration/Amount (%)	72.5	87.2	81.3

Standard deviations for biomass Chl-*a* are indicated inparentheses.

Assuming the lower limit of the phytoplankton contribution to be accurate, the clam population would tend toward consuming almost all of (or more than) the total MPB available in the tidal-flat area each day (Table 2). Resuspended primarily produced organic particles (i.e. those originating from MPB) in tidal flats are considered to be important food sources for benthic animals [43], and their lateral transport to the habitat of suspension-feeding benthic bivalves (*R. philippinarum* and *Mactra veneriformis* Reeve 1854) is thought to be essential to sustain the high secondary productivity of these bivalves [9]. In the inner part of Ariake Bay, resuspended MPB was supplied to the benthic community on tidal flats and provided an additional source of food in addition to that found in the subtidal region, thus increasing the food availability by 50% [44]. In the present study, the abundant MPB in the subtidal area (where MPB are approximately 18 times more abundant than in the tidal flat area) appear to have been a key clam food source, particularly in its resuspended form; in this manner, MPB could have sustained the high secondary productivity of the clam.

In the muddy tidal flats of Ariake Bay, southern Japan, a certain portion of the MPB typically becomes resuspended during high tide, thus contribution to the total biomass in the water column [13]. In the central part of the area investigated in the present study, resuspension of MPB due to the tidal cycle has been estimated to contribute up to 74% of water column Chl-*a* [45]. Therefore, the biomass of phytoplankton has likely been somewhat overestimated in the present study owing to the lack of consideration of resusupended MPB.

Interestingly, the contribution of *U. pertus* for the extracted subset to the clam diet (based on the amount of phytoplankton) was found to be 12–28% (Figure 8c). Previously, clam feeding experiments have demonstrated that the clam can filtrate a maximum particle size of 200 µm [40]. Furthermore, the hard clam *Mercenaria mercenaria* L. 1758, which is in the same family (Veneridae) as *R. philippinarum*, can filtrate aggregates up to approximately 1 mm in diameter [46]. These previous studies suggest that seaweeds must be broken down into fine particles (i.e. less than 1 mm diameter) to be filtrated. During this process, the numerous bacteria that are attached to the debris form a community and act as a further food source for the clam.

The results of the present study suggest a clear relationship between the isotopic signature and food demand of the clam population. The contribution of MPB was unclear before the extraction; however, the final results, which are revised with respect to the combination of food available and the total abundance of primary producers in the lagoon, indicate that MPB are likely the main food source for the clam population in this environment. The dense patches of clams with high productivity (130 g C m^−2^ yr^−1^) are likely sustained by the supply of particulate organic matter derived from MPB (35–64%) in the subtidal area, where the standing stock of MPB is approximately 18 times that for the tidal flats.

## Supporting Information

Figure S1 **Seasonal variations of macro-benthic biomass (in kgWW m^–2^) at each tidal flat station.**
(TIF)
