# Supplementary figures and images for: Food Sources for Ruditapes philippinarum in a Coastal Lagoon Determined by Mass Balance and Stable Isotope Approaches
Source: PLoS One. 2014 Jan 28;9(1):e86732. doi: 10.1371/journal.pone.0086732 (PMC3904936; doi:10.1371/journal.pone.0086732)

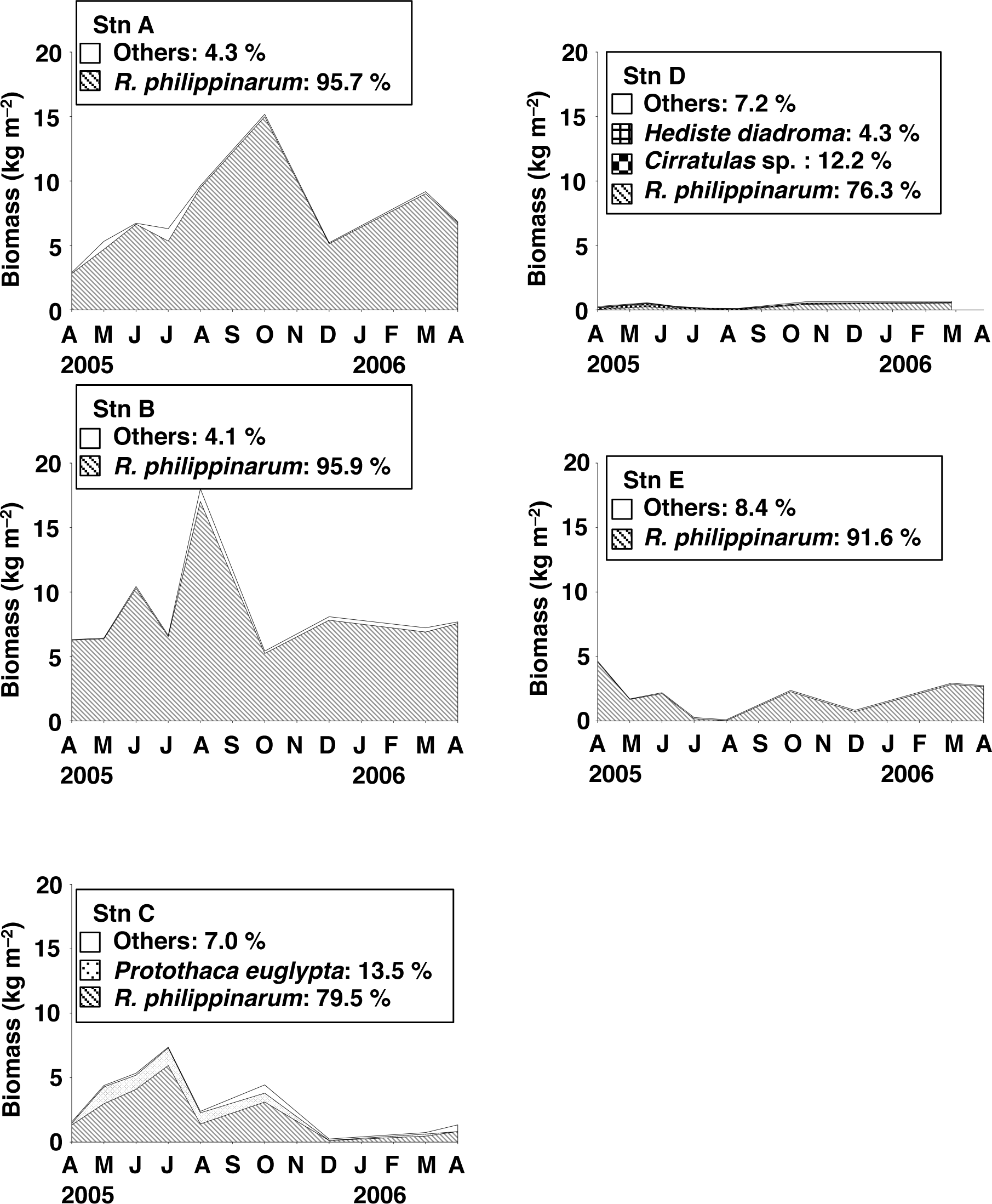

Supplement: Figure S1 — Seasonal variations of macro-benthic biomass (in kgWW m–2) at each tidal flat station. (TIF) [file pone.0086732.s001.tif]
